# Review of human pegivirus: Prevalence, transmission, pathogenesis, and clinical implication

**DOI:** 10.1080/21505594.2022.2029328

**Published:** 2022-02-08

**Authors:** Yaqi Yu, Zhenzhou Wan, Jian-Hua Wang, Xianguang Yang, Chiyu Zhang

**Affiliations:** a College of Life Sciences, Henan Normal University, Xinxiang, China; b Shanghai Public Health Clinical Center, Fudan University, Shanghai, China; c Medical Laboratory of Taizhou Fourth People’s Hospital, Taizhou, China; d Guangzhou Institutes of Biomedicine and Health, Chinese Academy of Sciences, Guangzhou, China

**Keywords:** Human pegivirus, prevalence, pathogenesis, human immunodeficiency virus type-1, hepatitis C virus

## Abstract

Human pegivirus (HPgV-1), previously known as GB virus C (GBV-C) or hepatitis G virus (HGV), is a single-stranded positive RNA virus belonging to the genus *Pegivirus* of the *Flaviviridae* family. It is transmitted by percutaneous injuries (PIs), contaminated blood and/or blood products, sexual contact, and vertical mother-to-child transmission. It is widely prevalent in general population, especially in high-risk groups. HPgV-1 viremia is typically cleared within the first 1–2 years of infection in most healthy individuals, but may persist for longer periods of time in immunocompromised individuals and/or those co-infected by other viruses. A large body of evidences indicate that HPgV-1 persistent infection has a beneficial clinical effect on many infectious diseases, such as acquired immunodeficiency syndrome (AIDS) and hepatitis C. The beneficial effects seem to be related to a significant reduction of immune activation, and/or the inhabitation of co-infected viruses (e.g. HIV-1). HPgV-1 has a broad cellular tropism for lymphoid and myeloid cells, and preferentially replicates in bone marrow and spleen without cytopathic effect, implying a therapeutic potential. The paper aims to summarize the natural history, prevalence and distribution characteristics, and pathogenesis of HPgV-1, and discuss its association with other human viral diseases, and potential use in therapy as a biovaccine or viral vector.

## Introduction

Human pegivirus (HPgV-1) is a spherical enveloped virus of about 50 nm in diameter [1]. It belongs to the genus *Pegivirus* of the family *Flaviviridae* and has a 9.4 kb positive-sense single-strand RNA genome that is organized similar to hepatitis C virus (HCV) [2,3]. HPgV included type 1 (HPgV-1) and type 2 (HPgV-2). HPgV-1 can cause persistent infection, but is not associated with hepatitis and other obvious clinical symptoms or diseases in healthy people [2]. In particular, a large number of studies have shown that HPgV-1 persistent infection slows the disease progression caused by human immunodeficiency virus type 1 (HIV-1) and/or other viruses and improves the survival of patients, suggesting that HPgV-1 infection plays a beneficial role when co-infected with other viruses [4–7,]. Currently, the natural history, pathogenic mechanisms, and potential impact of HPgV-1 on human health remain to be seen. In this paper, we summarize the history, prevalence and pathogenesis of HPgV-1, and discuss its relationship with other viral diseases, and the possibility of HPgV-1 as therapeutic tools or viral vectors.

## Discovery of pegivirus

HPgV-1 was formerly known as GB virus type C (GBV-C) or hepatitis G virus (HGV). The abbreviation “GB” came from a surgeon with acute hepatitis. In 1967, serum from the surgeon was experimentally inoculated into tamarins, and resulted in hepatitis in tamarins [3,6]. Therefore, the presence of a new unknown virus that causes hepatitis was predicted. Until 1995, two new RNA viruses were identified from tamarins that received inoculation of GB passage and developed hepatitis. Because the two viruses belong to the family *Flaviviridae*, and are different from the previously identified hepatitis A-E viruses, they were named as GB virus A (GBV-A) and GB virus B (GBV-B) [7]. In the same year, another novel RNA virus was identified in the serum of non-AE hepatitis patients from West Africa. The virus had 53% to 59% similarity to GBV-A and GBV-B nucleic acid sequences, respectively, and approximately 47% homology to HCV sequences. Based on sequence homology and phylogenetic analysis, the virus was classified as a new member of the *Flaviviridae* family and named as GBV-C [5,8]. In 1996, HGV was identified from a patient with chronic hepatitis [9]. Because HGV is closely genetically related to GBV-C, rather than GBV-A and GBV-B, GBV-C and HGV represent different isolates of the same virus species. GBV-C and HGV initially were believed to be associated with non-AE hepatitis in human [5–13]. In 2010, GBV-D was identified and described from free-ranging bats [10]. It shares about 50% identity to GBV-A and GBV-C at the amino acid level, and represents a distinct species within the family *Flaviviridae* [10].

**Figure 1.**
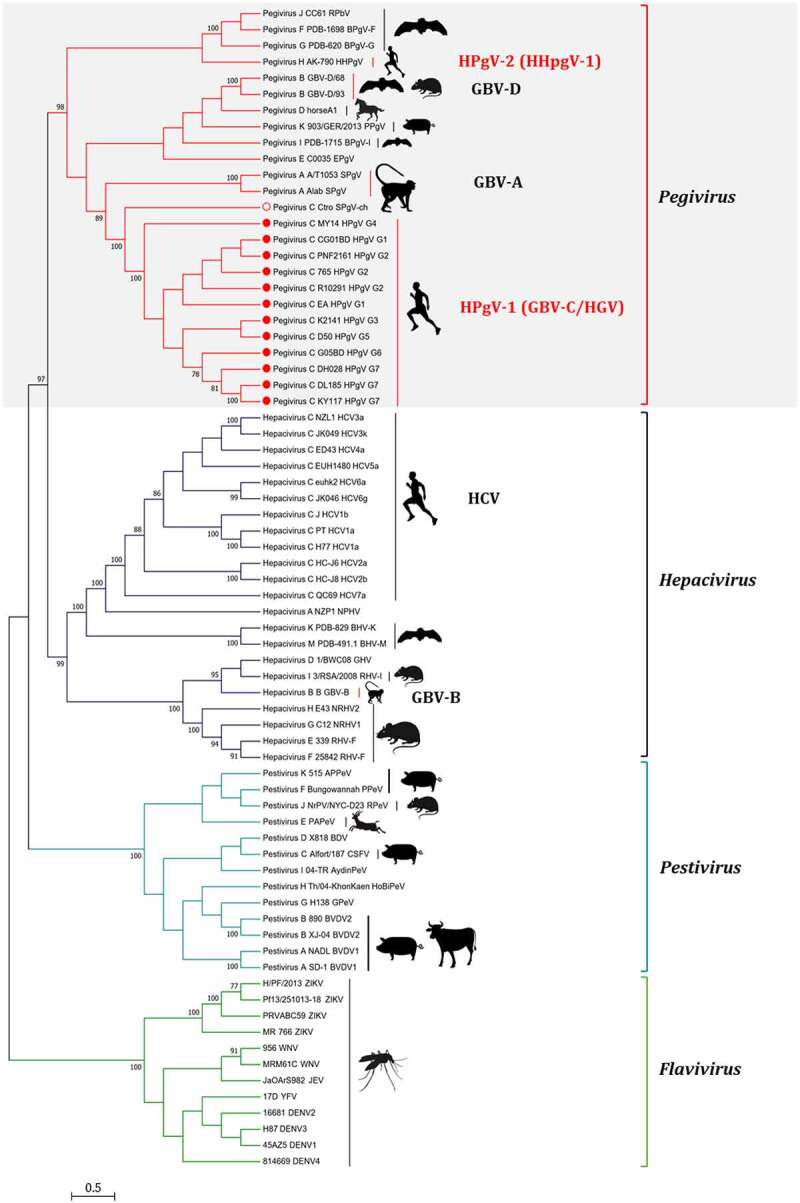
Phylogenetic relationship of pegivirus. The phylogenetic tree was constructed based on RdRp gene sequences of selected *Flaviviridae* members using the maximum-likelihood (ML) method (MEGA 7.0.26). Four genera are classified in the *Flaviviridae* family. The main hosts of these viruses are also shown in the figure. The red branches highlight the members of the genus *Pegivirus.*

Among GB viruses, only GBV-B was found to cause hepatitis, and was assigned to the genus *Hepacivirus* (Figure 1) [11,12]. GBV-C and other two viruses (GBV-A and GBV-D) were later found not to be associated with hepatitis. In 2011, Stapleton et al. assigned GBV-C, GBV-A, and GBV-D to the fourth genus of the family *Flaviviridae* according to their phylogenetic relationships, genome organization, and pathogenic features (Figure 1) [3]. The new genus was named as *Pegivirus* (pe, persistent; g, GB, or G). Mammals are the main hosts of pegiviruses, including primates [13–18], horses [19–22], bats, and rodents [10,23–25]. Recent studies showed that pegiviruses can also infect non-mammals, such as geese [26,27], illustrating a wide range of hosts. Because GBV-C/HGV infects human beings, it was renamed as human pegivirus type 1 (HPgV-1). The second human pegivirus (HPgV-2, also known as HHpgV-1) was firstly identified from blood transfusion recipients in the US in 2015 [28,29], and later detected in other countries (e.g. China [30,31], Vietnam [32], Cameroon [33]) (Figure 1). Figure 1 shows the phylogenetic relationship of pegiviruses.

## Genome organization and protein products of HPgV-1

**Figure 2.**
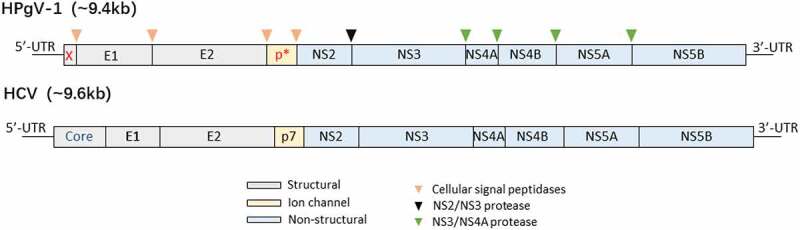
Genome organization of HPgV-1 and HCV. The genome encodes a single pre-polyprotein that is cleaved into mature viral proteins after co-translation and post-translation. Compared to HCV, HPgV-1 genome encodes two additional predicted proteins (protein X at upstream of E1, and protein p* between E2 and NS2), but does not encode a core protein that is an RNA-binding protein and forms the virion nucleocapsid.

Like other members of the family *Flaviviridae*, HPgV-1 genome encodes an open reading frame (ORF) that is translated into a single pre-polyprotein consisting of approximately 3000 amino acid residues (Figure 2) [3,8]. The coding region is flanked by long 5’ and 3’ untranslated regions (UTRs). The 5’-UTR contains an internal ribosome entry site (IRES), which recruits ribosomes to guide viral mRNA translation [3,34]. The pre-polyprotein is further cleaved into two structural proteins (envelope proteins E1 and E2) and six non-structural proteins (NS2, NS3, NS4A, NS4B, NS5A, and NS5B) by cellular and viral proteases (Figure 2).

Because of sharing similar genome organization and homologous genes to HCV [3], HPgV-1 is believed to have similar life cycle to HCV, including i. viral attachment and entry; ii. endocytosis; iii. fusion and uncoating; iv. translation and polyprotein processing; v. RNA replication; vi. virion assembly; vii. virion release [35]. Its proteins are also predicted to have similar functions with their counterparts in HCV [36–39]. Compared with HCV, the encoding region for a core protein is not identified for HPgV-1 (Figure 2) [3,40]. However, a basic protein is predicted at upstream of the signalase site before E1 in HPgV-1 genome [40]. This protein may participate in RNA packaging during virion assembly. Another additional protein (p*) is an about 6 kDa protein analogous to the HCV p7. Structural proteins E1 and E2 are envelope glycoproteins [36]. They are released from pre-polyprotein via enzymatic hydrolysis by a host signal peptidase [3,36]. By forming heterodimers on the surface of viral particles, they participate in viral assembly and are responsible for virus entry. E2 glycoprotein is responsible for the binding of the virus to cell receptors, which induces membrane fusion and promotes the entry of HPgV-1 into host cells [36,41]. E2 glycoprotein possesses immunogenicity and induces humoral immune response [2,42,43]. Furthermore, E2 glycoprotein interacts with co-infected viruses (e.g. HIV-1) and host proteins, and further participates in the regulation of host immune activation [44,45]. It alters IL-2-signaling pathways by reducing TCR-induced IL-2 production to inhibit the T-lymphocyte activation, and inhibits the IL-12 signaling pathway to reduce the proliferation of NK cell [46,47].

NS2, NS3, and NS4A are responsible for the cleavage of non-structural proteins [41,48,49]. The cleavage of NS2/NS3 is mediated by NS2 protease, and the cleavage of other NS proteins is mediated by NS3 protease with NS4A as a cofactor [49,50]. NS4B is a highly hydrophobic protein that may be involved in the formation of membranous structures supporting RNA replication. NS5A is known as a cytoplasmic phosphorylated protein that may participate in and regulate RNA replication [48]. NS5B is a RNA-dependent RNA polymerase that is responsible for genome replication of HPgV-1 [8,49]. HPgV-1 proteins and their functions are summarized in Table 1.

**Table 1.** HPgV-1 proteins and their functions

Protein	Function
E1	Envelope glycoproteins
E2	Envelope glycoproteins, receptor binding
p7-like	Similar in size to HCV p7
NS2	Component of the NS2-3 protease, mediating cleavage at the NS2/NS3 junction
NS3	Protease, mediating the cleavage of NS proteins, C-terminal NTPase and helicase
NS4A	Cofactor for NS3-mediated cleavages of NS proteins
NS4B	Membrane alteration inducer
NS5A	Multifunctional phosphoprotein
NS5B	RNA-dependent RNA polymerase, genomic RNA replication

Note: The functions of some HPgV-1 proteins are predicted according to their counterparts in HCV.

## Prevalence and distribution

**Figure 3.**
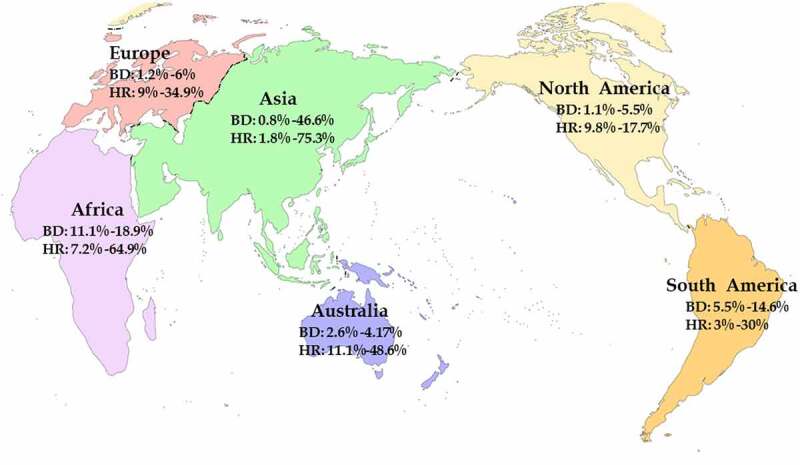
Global prevalence and distribution of HPgV-1. BD: blood donors; HR: high-risk population mainly including IDUs, CSWs, and MSM.

HPgV-1 has a high global prevalence. About one-sixth of the global population was estimated to be sero-positive for HPgV-1, and approximately 750 million people had viremia [2,3,51,52]. The at-risk population had substantially higher prevalence of HPgV-1 than the general population, and the prevalence of HPgV-1 varied considerably in different countries/regions of the world (Figure 3). In the general population and healthy blood donors, HPgV-1 prevalence ranged from 0.8% to 44.6%, while in the at-risk population, the prevalence rate ranged from 1.8% to 75.3% [53–96]. HPgV-1 has a higher prevalence in the developing world than in the developed world (Figure 3). For example, HPgV-1 prevalence in healthy blood donors was 0.8–46.6% in the developing world (e.g. Asia, Africa, and South America), while the rate was 1.1–6% in the developed world (e.g. North America, Europe, and Australia). Similar trend was also observed in the at-risk population with high HPgV prevalence (1.8–75.3%) in the developing world, but relatively low prevalence (9–48.6%) in the developed world. Geographic difference in HPgV-1 prevalence was believed to be associated with the socio-economic situation of a country/region, which reflects the income and welfare levels of local people, and affects their medical and health conditions [51]. People with lower income and welfare levels appeared to have a higher risk of HPgV-1 infection than those with higher income and welfare levels since the former more likely participate in illegal or paid blood donation and reuse unsterilized needles and/or contaminated instruments.

On the other hand, HPgV-1 prevalence appears to have obvious genotypic and geographical characteristics. HPgV-1 genotypes 1 and 2 are mainly distributed in Africa [54,55,78]; genotype 2 is more prevalent in Europe [79,80]; genotype 3 is prevalent in Asian countries and South America [81–84]; genotype 4 and 5 remains dominant in Philippines and other countries in Southeast Asia [85,86]; genotype 6 is circulating in Indonesia [87]. Genotype 7 was recently found in Yunnan Province of China, and some other Asian countries, such as Qatar [88,89]. The difference in the distribution of HPgV-1 genotypes might be associated with origin, evolution, and transmission of these genotypes.

## Transmission and at-risk population of HPgV-1

Like HIV-1, HBV, and HCV, HPgV-1 is a blood-borne virus [2,90]. It is efficiently transmitted by percutaneous injuries (PIs) and blood transfusion, which explains why high proportion of HPgV-1 infection was found among healthy blood donors. Because of high-frequency exposure behavior, intravenous drug users (IDUs) are the major high-risk group for HPgV-1 infection, and have very high positive rate for this virus (Figure 3). Furthermore, people who received acupuncture were found to have significantly higher prevalence of HPgV-1 (16.5%) than those who never received acupuncture (9.4%) [91], implying that acupuncture increases the risk of HPgV-1 infection.

Apart from occupational exposure to PIs, and contaminated blood and/or blood components, HPgV-1 can also be transmitted by sexual contact (including heterosexual and homosexual contacts) and vertical mother-to-child transmission [87,89–94]. Commercial sex workers (CSWs) and men who have sex with men (MSM) are also the major high-risk groups for HPgV-1 infection. Because of sharing the same transmission routes with HIV-1, HCV, and HBV, high proportion (3.2–47.9%) of HPgV-1 co-infection was often reported in the individuals who are positive for the above-mentioned viruses [95–104].

### Blood donors

HPgV-1 prevalence in blood donors varied largely in different countries/regions (0.8–46.6%) (Figure 3). The vast majority of the studies reported HPgV-1 prevalence less than 5% in blood donors, while few studies showed higher HPgV-1 prevalence (>10%) in some countries/regions (e.g. India [105,106], China [107–122], Kuwait [108]). The global prevalence of HPgV-1 was estimated to be 3.1% in blood donors [51]. The pooled prevalence of HPgV-1 was 1.7% in North America, 9.1% in South America, 2.3% in Europe, and 2.4% in Asia [51]. Based on 67,348 blood donors, HPgV-1 prevalence was estimated to be 3.3% in China [110]. Currently, HPgV-1 is not included in the routine blood donor screening test. The prevalence of HPgV-1 in general population and blood donors highlight the risk of post-transfusion infection even though HPgV-1 infection was largely believed to be benign. Concerns are being raised on whether screening for HPgV-1 should be included in the routine blood donor assay.

### IDUs

IDUs are the most important high-risk group for HPgV-1 infection, and have prevalence of 11.6–89.2% in different studies. HPgV-1 viremia was more common among IDUs compared to healthy volunteers [79]. Based on 3779 IDUs from different studies, the pooled prevalence of HPgV-1 was estimated to be 33.6% [79,89,92,101,111–138]. Furthermore, HPgV-1 prevalence among IDUs appeared to be higher in developed world than those in developing world. For example, few studies showed that HPgV-1 prevalence among IDUs reached 89.2% in North America [117], 41.9% in Australia [113,121], while in Africa and South America, the pooled prevalence was 20.8% and 25.8%, respectively. In Asia and Europe, the pooled prevalence was 32% and 34%, respectively.

Because of frequent needle sharing behavior, co-infection of HPgV-1 with HIV-1, HBV, and/or HCV was very common among IDUs. The co-infection rate ranged from 11.6% to 85.8% [101,111,114,121,122,129,131,133,137–139]. In particular, the prevalence of triple infection with HPgV-1, HIV-1, and HCV was often higher than that of dual infection by HPgV-1 and HIV-1 or HCV [111,132,133].

### CSWs and MSM

Sexual transmission routes of HPgV-1 include heterosexual and homosexual transmission. CSWs and MSM are the most predominant high-risk groups for heterosexual and homosexual infection of HPgV-1, respectively. HPgV-1 prevalence varied from 0% to 35.5% among CSWs and from 12.5% to 36.2% among MSM [52,89,92,120,121,126,128,131–133,135,136,138,140–143]. The pooled prevalence of HPgV-1 was 17.3% and 19.7% in CSWs and MSM, respectively, suggesting similar transmission risk of HPgV-1 among the two high-risk groups. Importantly, the worst-hit continent of HPgV-1 prevalence was CSWs in Asia (17.9%) and MSM in Australia (36.2%), respectively.

## Pathogenesis

The pathogenicity of HPgV-1 remains controversial. A large number of epidemiological and clinical studies did not support an association of HPgV-1 infection with any known clinical diseases (reviewed in [4,144,145]). Although the virus was detected in the saliva and serum of healthy people and replicates *in vivo* at high titer, neither obvious clinical symptoms were observed, nor significant immune activation in any cell types was detected [146,147]. Approximately 80% of healthy people or immune competent individuals spontaneously clear viraemia within 2 years of HPgV-1 infection [2,3,117,148]. However, in immunocompromised individuals and/or individuals with other infectious diseases, HPgV-1 viraemia can persist for up to decades [2]. The maintenance of persistent infection may be ascribed to the ability of HPgV-1 to avoid immune recognition and T cells immune activation [36,149]. HPgV-1 E2 glycoprotein is believed to contain T cell receptor-inhibitory motifs, and contributes to viral persistence by reducing T cells immune activation [149]. On the other hand, HPgV-1 does not induce broad antibody responses. The specific antibody response appears to be restricted to E2 [2,3,42,117]. Anti-E2 antibody is associated with the clearance of HPgV-1 viraemia, and can prevent HPgV-1 reinfection [2,43,150].

HPgV-1 is frequently co-infected with other blood-borne viruses, such as HIV-1 and HCV. HPgV-1 persistent infection inhibits abnormal and excessive immune activation in patient co-infected with HIV-1, HCV, or EBOV, and often shows beneficial clinical effects in these patients [4,144,145]. In particular, HPgV-1 infection slows disease progression and prolong survival time of HIV-1 infected individuals by directly inhibiting HIV-1 infection and replication, and/or reducing immune activation of T lymphocytes [151–158].

Although most HPgV-1 infections are self-limited, few immunocompromised individuals with HPgV-1 infection developed lymphoma [159–164]. In 2018, Fama et al. reported that HPgV-1 infection was closely associated with the overall risk of lymphoma [165]. The association was observed for almost every major lymphoma subtype except chronic lymphomatous leukemia (CLL)/small lymphocytic lymphoma (SLL) and Hodgkin’s lymphoma (HL). A recent meta-analysis supported the positive association of HPgV-1 persistent infection with lymphoma risk [166]. HPgV-1 is a lymphotropic virus that causes persistent infection in both T and B lymphocytes [2,167]. Persistent HPgV-1 infection may induce DNA mutations and potentially malignant transformation in lymphocytes, which promote the development of lymphoma [165,166]. The possible causal relationship between HPgV-1 viremia and lymphoma risk suggests that HPgV-1 may be a risk marker and a potential therapeutic target for lymphoma.

Furthermore, Balcom et al. reported two cases of HPgV-1 related fatal brain leukocyte encephalitis, in which lymphocytic infiltration and gliosis were detected in the brain tissue, suggesting neurotropism of HPgV-1 [168]. The neural cell tropism of HPgV-1 was supported by another recent study that showed that HPgV-1 infects specific nerve cells in the human brain, such as astrocytes and microglia [169]. By inhibiting antiviral signaling pathways, HPgV-1 can establish persistent infection and promote the development of neurological diseases [169].

Given the potential association of HPgV-1 infection with the lymphoma risk [165,166], development of anti-HPgV-1 small molecule drugs might be beneficial for the treatment and prophylaxis of lymphoma. However, it is very time- and resource-consuming for the development of efficient antiviral small molecule drugs, which needs a suitable *in vitro* culture system for virus growth. Currently, there was no suitable culture system of HPgV-1 production (reviewed in [2]). Therefore, repurposing of existing anti-drugs may be an alternative strategy. One exciting development in antiviral researches is the development of direct-acting antivirals (DAAs) against HCV [170–173]. DAAs target the NS3/4A protease, the NS5A protein, and the NS5B polymerase of HCV, and cure HCV infection in over 90% of patients [172]. Despite the fact that HPgV-1 shares homogenous genes and has close epidemiological association with HCV, DAAs seem not to inhibit HPgV [174]. In view of the fact that HPgV-1 infection is benign in healthy individuals, whether it is necessary to develop anti-HPgV-1 therapeutic drugs deserves to be cautiously assessed.

## HPgV-1 co-infection and human diseases

HPgV-1 has a high co-infection rate with other human viruses such as HIV-1 and HCV. HPgV-1 persistent infection can lead to significant improvements in clinical parameters and outcomes in patients co-infected with other viruses (Table 2), showing a beneficial effect on other viral diseases to some extent [4,144]. The beneficial outcome of HPgV-1 persistent infection was mainly associated with the inhibition or reduction of abnormal and excessive immune activation, especially the immune activation of T lymphocytes [4,144,145].

**Table 2.** HPgV-1 prevalence in persons co-infected with HIV-1 or HCV or EBOV and clinical outcomes

HPgV-1 co-infected cohorts	HPgV-1 prevalence	Clinical outcomes
**HIV-1 infected**	5−47.9%	Higher survival, CD4 cell counts, and CD4+/CD8+ ratio Lower HIV-1 viral loads, and T-cell activation Slower progression to AIDS Decreased levels of cytokines and chemokines Down-regulation of CCR5 and CXCR4 expression Improved response to HAART Superior quality of life
**HIV-1/HCV co-infected^a^**	11.8−37.2%	Reduction in cirrhosis, hepatic fibrosis and inflammation Down-regulation of LCK and DOK2 expression Lower ALT and AST levels
**EBOV infected**	26.5% **^b^**	Higher survival

**a**: HPgV-1 infection does not show significant beneficial effect for HCV-mono-infected individuals. Furthermore, the data and clinical findings are mostly based on patients with HIV-1/HCV/HPgV-1 triple co-infection. **b**: Data from one report.

### Co-infection with HIV-1

Approximately 5–47.9% HIV-1 infected individuals were co-infected with HPgV-1 [55,89,95–100,151,153–156,175–185]. People who co-infected with HIV-1/HPgV-1 generally had relatively slower disease progression of AIDS and prolonged survival [89,153,155,156,181]. CD4 + T cell count and HIV-1 viral load are two crucial predictors of HIV/AIDS disease progression, and are used to determine the initiation and to evaluate the efficacy of highly active antiretroviral therapy (HAART) [186,187]. A large number of studies revealed that HPgV-1 viral load is significantly positively correlated to CD4 + T cell number, but negatively correlated to HIV-1 viral load [97,98,151,153,154,175,176,178–182,188–191] (Table 2). These findings indicate that HPgV-1 persistent infection is associated with a beneficial effect on HIV/AIDS.

There are diverse mechanisms involving in the beneficial effect of HPgV-1 co-infection on HIV-1 disease progression. First, HPgV-1 infection reduces surface expression of chemokine receptors CCR5 and CXCR4, both of which serve as co-receptors for HIV-1 entry into host cells (including CD4 + T cells, macrophages, DC cells) [183,192–195]. On the other hand, HPgV-1 E2 glycoprotein and NS5A protein can up-regulate the productions of the CCR5 ligands (e.g. RANTES, MIP-1α, and MIP-1β) and the CXCR4 ligand SDF-1, respectively [97,190,193,196]. Down-regulation of HIV-1 co-receptors and increased release of their ligands inhibit HIV-1 entry and reduce viral cell-to-cell transmission. Second, HPgV-1 E2 glycoprotein can reduce the production of mature capsid protein P24 and matrix protein P17 by inhibiting the processing of HIV-1 Gap precursor (P55), and thereby inhibit HIV-1 assembly [144,145,197,198]. Third, persistent immune activation and decreased Th1/Th2 cytokine ratio in HIV-1 infection are associated with rapid progression to AIDS. HPgV-1 co-infection reduces HIV-1-mediated activation of T, B, and/or NK cells, and contributes to the maintenance of balance between T-helper 1 (Th1) cytokines and Th2 cytokines, which delay the development of AIDS [199–202]. For example, HPgV-1 E2 glycoprotein inhibits T cell activation by reducing TCR-induced IL-2 production and altering IL-2 signaling pathways [152,156,202–205]. Furthermore, HPgV-1 infection is associated with an increase of CD4 and CD8 double-negative T cells (CD4-CD8-CD3+), which also contribute to reduction of immune activation and maintenance of immune homeostasis, further improving the survival of HIV-1 infected individuals [144,184,206]. Fourth, HPgV-1 E2 glycoprotein can induce antibodies to neutralize and precipitate diverse HIV-1 isolates possibly by cross-reaction with a cellular antigen on HIV-1 particles [45]. Fifth, HPgV-1 co-infection was reported to control HIV-1 replication by activating the endogenous interferon system, and to reduce Fas-mediated apoptosis of CD4 + T cells by down-regulating Fas expression [207]. Furthermore, HPgV-1 co-infection appeared to improve the response to HAART in HIV-infected individuals and the duration of HAART did not reduce HPgV-1 viremia [178,208].

### Co-infection with HCV

HPgV-1 infection was closely related to HCV infection because of sharing the same transmission routes. About 11.8–37.2% HCV-infected individuals were co-infected with HPgV-1 [101–103,209–212]. Recent studies showed that HCV-infected individuals were also found to have a high proportion of HPgV-2 infection [30–32,174,213,214]. HPgV-1 infection was found to be associated with a significant reduction in the severity of HCV-related liver disease in HCV/HIV-1-co-infected patients, showing a beneficial influence [101,103,210]. In HCV/HIV-1-co-infected patients, HPgV-1 persistent infection remarkably decreases AST and ALT levels by down-regulating some crucial genes from intra-hepatic T-cell signal transduction, and then significantly improves chronic hepatitis C-related liver injury and reduces the incidence of hepatopathy (Table 2) [101,210]. These genes include LCK, DOK2, interleukin 2 receptor gamma (IL2R-γ), and cyclin D3 (CCND3), and are closely associated with T-cell receptor complex (TCR) [210]. However, a similar beneficial influence of HPgV-1 infection was not observed in HCV mono-infected patients [103]. The possible reason for this difference is that HPgV-1 is also a lymphotropic virus that may interact with HIV-1 by infecting the same cells, but not with HCV because of different cell targets.

### Co-infection with Ebola virus

Ebola virus (EBOV) is an aggressive virus that causes highly lethal Ebola hemorrhagic fever (EHF) on humans and non-human primates. In Sierra Leone, Liberia and Guinea, the worst-hit areas by Ebola epidemic, about 11.1–18.9% of healthy individuals were infected by HPgV-1 (Figure 3). In a retrospective study that analyzed previous deep-sequencing data, 13 (26.5%) of 49 EBOV-infected individuals were found to be co-infected with HPgV-1 [215]. The survival rate of HPgV-1 co-infected Ebola patients was 53.8%, significantly higher than that (22.2%) of HPgV-1 negative Ebola patients, suggesting that HPgV-1 co-infection may attenuate the pathogenicity of EBOV [215]. The beneficial effect of HPgV-1 co-infection on Ebola patient might be also associated with reduced proinflammatory cytokines production and excessive T-cell activation.

## Virus isolation and animal models

### Cell tropism and host range

Because HPgV-1 was first identified from patients with acute or chronic non-A-E hepatitis, it was initially considered as a hepatotropic virus [3,6,5,8,9,37]. However, subsequent evidences did not support an association of HPgV-1 infection with either acute and/or chronic hepatitis. In particular, HPgV-1 RNA was found to be more frequently detected in circulating lymphocytes, but not or in a very low level in liver biopsies of infected people [2,216–218]. Furthermore, HPgV-1 RNA level remained relatively stable in patients with pre-transplantation HPgV-1 infection after liver transplantation, while HCV RNA level increased steady in patients with chronic hepatitis C after liver transplantation [219]. These evidences suggest that HPgV-1 is lymphotropic, rather than hepatotropic.

HPgV-1 RNA was detected in multiple lineages of peripheral blood mononuclear cells (PBMCs, including T lymphocytes, B lymphocytes, NK cells, and monocytes), indicating a wide tropism [167,220–222]. However, HPgV-1 negative-strand RNA, the marker of viral RNA replication, was preferentially detected in bone marrow and spleen, but less in PBMCs, suggesting that progenitor haematopoietic stem cell (HSC) may also be the primary target of HPgV-1 infection (reviewed in [2]). The presence of HPgV-1 in PBMCs indicates that the virus persists and replicates during and following subsequent lymphocyte maturation [2,218,220,223,224].

Old world primates are believed to be the natural hosts of HPgV-1 [18,225]. Apart from humans, HPgV-1 can also infect chimpanzees and macaques [225,226]. Whether other primates and/or animals are also susceptible to HPgV-1 infection remains to be determined.

### In vitro culture of HPgV-1

Establishment of an *in vitro* cell culture system is crucial for studying the biological characteristics and molecular mechanisms of HPgV-1, as well as developing strategies for prophylactic and therapeutic interventions. Development of an efficient cell culture system depends on permissive cells (primary cells or cell lines) supporting infection and production of infectious virion, and a virus or its infectious clone capable of replicating and assembling virion in permissive cells.

As a lymphotropic virus, HPgV-1 extensively exists in multiple lineages of PBMCs [2,167], and PBMCs from HPgV-1 infected people were demonstrated to transfer the virus to primary PBMCs of healthy individuals *in vitro* [220,221]. Serum from HPgV-1-infected individuals was also demonstrated to establish infection in PBMCs *in vitro* [167]. Using primary PBMCs, *in vitro* HPgV-1 culture systems have been previously established, and the culture could be maintained up to 35 days [220,221]. However, HPgV-1 replication appears to be very limited in PBMCs. The virus can be poorly produced *in vitro* PBMCs culture system, and average less than 10 HPgV-1 genomic copies can be detected among per 100 PBMCs [167,223]. These imply that only a very small proportion of PBMCs support HPgV-1 replication, or there are some potential cellular restriction factors to inhibit HPgV-1 replication in PBMCs [227]. On the other hand, because cellular receptors for HPgV-1 infection remain unknown, the permissive cell lines supporting HPgV-1 infection and replication need to be determined [2].

In recent years, reverse genetics systems have been developed and provide powerful tools to recover some uncultivated viruses [228]. Using the systems, infectious clones of some emerging viruses, including HCV [229,230], Zika virus, and dengue virus from the *Flaviviridae* family [231–234], and the newly emerging SARS-CoV-2 [235–237], have been constructed. Although currently the infectious clone of HPgV-1 was not available, two full-length cDNA clones of HPgV-1 were previously constructed and their *in vitro* full-length RNA transcripts were proved to be infectious in primary CD4 + T cells [238] and in macaques (Macaca mulatta) [239]. In view of sharing similar genome organization and homogenous genes to HCV, the success in the development of efficient cell culture systems for HCV and other *Flaviviridae* viruses [229,230] and availability of increasing number of complete HPgV-1 genome sequences provide avenues for the development of HPgV-1 infectious clones using reverse genetics tools in the future.

### Animal models

The lack of appropriate animal models for HPgV-1 infection limits the understanding of its pathogenesis. Non-human primates (NHPs) are considered to be the ideal animal models for viral diseases since they are closely genetically related to humans than other animals. As the most widely used animal models for viral diseases such as HIV/AIDS, macaques and chimpanzees are considered as primary animal models of HPgV-1 infection since they might be susceptible to HPgV-1 infection [18,225,226,239–241]. For ethical and financial reasons, macaques are preferred to be used for the NHP model of HPgV-1. However, macaques often failed to be experimentally infected with HPgV-1 [3].

Fortunately, some simian pegiviruses (SPgV) that are closely genetially related to HPgV-1 were recently identified and characterized from some old world monkey species (e.g. red colobus monkeys, red-tailed guenons, and olive baboon) [18]. Using a SPgV strain isolated from yellow baboons in Mikumi National Park, Tanzania, a macaque model of HPgV-1 infection was recently established [225]. The SPgV-infected macaques showed similar clinical characteristics (e.g. persistent infection, high-titer viremia, and lack of obvious pathogenic symptoms) to HPgV-1 infected humans. In this model, bone marrow and spleen were further confirmed to be the predominant tissues for HPgV-1 replication and production [225].

On the other hand, development of NHP models for human infectious diseases was largely limited by extremely high cost, difficulty to reach sufficient sample size, as well as raising ethical concerns of experimentation on NHPs. As the most widely used small animal models, humanized mice might represent a rapid, convenient, and promising direction for the development of animal models for HPgV-1 infection and other human viral diseases due to their rapid reproductive capacity, clear genetic background, and well-defined immune systems [242].

## Potential use of HPgV-1 in therapy

As a non-pathogenic virus, a large number of epidemiological and clinical studies demonstrated the protective effect of HPgV-1 persistent infection on HIV-1 infection, which was well supported by diverse molecular mechanisms, involving in the inhabitation of HIV-1 entry and replication and the suppression of immune activation [2,36,144]. Similar beneficial effect of HPgV-1 infection was also observed in Ebola patients and HIV-1/HCV co-infected patients [101,210,215]. These imply a high potential of HPgV-1 as a therapeutic bio-vaccine to be used in people living with HIV/AIDS in resource-limited regions where HAART is not common [243]. Furthermore, HPgV-1 was demonstrated to preferentially infect and replicate in HSC without cytopathic effect, implying another potential therapeutic application of HPgV-1 as viral vectors [218,224,225].

Therapeutic potential of HPgV-1 as a biovaccine was recently validated in a macaque model, in which the monkeys were sequentially infected by SPgV and simian immunodeficiency virus (SIV) [244]. In the model, the protective effect of SPgV was found to preferentially occur during the chronic phase of SIV infection [244]. In 2019, Greenhalgh and colleagues evaluated the feasibility of HPgV-1 as a biovaccine for HIV/AIDS [245]. Based on the epidemiological data of AIDS among MSM, they constructed a mathematical model to evaluate the potential impact of HPgV-1 biovaccination on AIDS-associated morbidity and mortality. They revealed that HPgV-1 biovaccination can effectively reduce the incidence of HIV/AIDS, AIDS-associated death and improve disability-adjusted life years (DALYs) of HIV-1 patients. Furthermore, the detrimental impact from HPgV-1 evolution was found to be very small under relatively high biovaccination rates (>12.5% annually) [245]. In fact, HPgV-1 is relatively evolutionarily conservative [246–248]. The SPgV-infected macaque model also supported an extremely low propensity of pegivirus to accumulate sequence variation [225]. In this model, about 1.5 variants were identified per 100 infection-days, and no consensus-level variants were detected, implying a very low risk of the HPgV-1 biovaccine strain evolving to pathogenic variants [225]. On the other hand, HPgV-1 co-infection seemed not to alter the evolution of HIV-1 [245]. Despite the significant progress in this field, it is still a long way for HPgV-1 to be used as a biovaccine to treat human infectious diseases. A major challenge is lack of an efficient *in vitro* culture system for HPgV-1 growth, which requires to first elucidate the cellular receptor of the virus and then develop appropriate (permissive) cell lines [2]. Furthermore, clinical trials to evaluate the effectiveness of HPgV-1 biovaccination in HIV-1-infected people are mandatory before regulatory approval and clinical application.

## Conclusion and future perspective

HPgV-1 is more like a non-pathogenic virus in the *Flaviviridae* family in spite of recent observation of a positive association of HPgV-1 viremia with increased risk of lymphoma in immunocompromised individuals. It is commonly prevalent in general population with extremely high proportions in high-risk groups such as IDUs and MSM. Persistent HPgV-1 infection slows the disease progression and improves the survival of individuals co-infected with HIV-1 and other pathogens by diverse molecular mechanisms, showing significant beneficial clinical effects. As a non-cytopathic lymphotropic virus that infects and preferentially replicates in bone marrow and spleen, HPgV-1 shows high therapeutic potential for infectious diseases as a biovaccine or a safe viral vector.

Although the promising results in HPgV-1 related epidemiological, experimental, and clinical studies, several underlying questions remain to be addressed. First, lack of an efficient *in vitro* culture system is a major barrier that limits the molecular biological mechanism researches of HPgV-1, including but not limited to the identification of cellular receptor of the virus, life cycle, persistence and clearance mechanism, viral interactions with the host immune system (interacting with immune cells and regulating immune activation), and with co-infected viruses (e.g. HIV-1, HCV, EBOV) or other pathogens (e.g. malaria) [249], as well as the development of suitable animal models and therapeutic biovaccine and/or vectors. Second, the association of HPgV-1 infection with the development of lymphoma, and even neurological diseases also need to be cautiously and systematically assessed. Furthermore, currently, COVID-19 is still the greatest threat to global public health. Whether there is a difference in the susceptibility to SARS-CoV-2 infection and the disease severity of COVID-19 between HPgV-1 infected and uninfected people deserves to be investigated.

## Data Availability

Data sharing is not applicable to this article as no new data were created or analyzed in this study.

## Abbreviations

AIDS: Acquired immunodeficiency syndrome
CCND3: Cyclin D3
CLL: Chronic lymphomatous leukemia
COVID-19: Corona virus disease 2019
CSWs: Commercial sex workers
DAAs: Direct-acting antivirals
DALYs: Disability-adjusted life years
EBOV: Ebola virus
EHF: Ebola hemorrhagic fever
GBV-A or -B or -C or -D: GB virus A or B or C or D
HAART: Highly active antiretroviral therapy
HCV: Hepatitis C virus
HGV: Hepatitis G virus
HIV-1: Human immunodeficiency virus type 1
HL: Hodgkin<apos;>s lymphoma
HPgV-1: Human pegivirus type 1
HPgV-2: Human pegivirus type 2 or Human hepegivirus type 1
HSC: Haematopoietic stem cell
IDUs: Intravenous drug users
IL2R-γ: Interleukin 2 receptor gamma
IRES: Internal ribosome entry site
MSM: Men who have sex with men
NHPs: Non-human primates
ORF: Open reading frame
PBMCs: Peripheral blood mononuclear cells
PIs: Percutaneous injuries
RdRp: RNA-dependent RNA polymerase
SARS-CoV-2: Severe acute respiratory syndrome coronavirus 2
SIV: Simian immunodeficiency virus
SLL: Small lymphocytic lymphoma
SPgV: Simian pegiviruses
TCR: T-cell receptor complex
Th1(or 2): T-helper 1 (or 2) cytokines
UTRs: Untranslated regions
DC: Dendritic cell
NK cell: Natural killer cell
